# The Short-term Effects of Temperature on Infectious Diarrhea among Children under 5 Years Old in Jiangsu, China: A Time-series Study (2015–2019)

**DOI:** 10.1007/s11596-021-2338-x

**Published:** 2021-04-20

**Authors:** Nan-nan Huang, Hao Zheng, Bin Li, Gao-qiang Fei, Zhen Ding, Jia-jia Wang, Xiao-bo Li

**Affiliations:** 1 Key Laboratory of Environmental Medicine Engineering, Ministry of Education, School of Public Health, Southeast University, Nanjing, 210009 China; 2 Department of Environmental Health, Jiangsu Provincial Center for Disease Control and Prevention, Nanjing, 210009 China; 3 Department of Toxicology and Sanitary Chemistry, School of Public Health, Capital Medical University, Beijing, 100069 China

**Keywords:** infectious diarrhea, incidence, meteorological factors, maximum temperature (Tmax), lag-effect

## Abstract

The association between meteorological factors and infectious diarrhea has been widely studied in many countries. However, investigation among children under 5 years old in Jiangsu, China remains quite limited. Data including infectious diarrhea cases among children under five years old and daily meteorological indexes in Jiangsu, China from 2015 to 2019 were collected. The lag-effects up to 21 days of daily maximum temperature (Tmax) on infectious diarrhea were explored using a quasi-Poisson regression with a distributed lag non-linear model (DLNM) approach. The cases number of infectious diarrhea was significantly associated with seasonal variation of meteorological factors, and the burden of disease mainly occurred among children aged 0–2 years old. Moreover, when the reference value was set at 16.7°C, Tmax had a significant lag-effect on cases of infectious diarrhea among children under 5 years old in Jiangsu Province, which was increased remarkably in cold weather with the highest risk at 8°C. The results of DLNM analysis implicated that the lag-effect of Tmax varied among the 13 cities in Jiangsu and had significant differences in 8 cities. The highest risk of Tmax was presented at 5 lag days in Huaian with a maximum RR of 1.18 (95% CI: 1.09, 1.29). Suzhou which had the highest number of diarrhea cases (15830 cases), had a maximum RR of 1.04 (95% CI:1.03, 1.05) on lag 15 days. Tmax is a considerable indicator to predict the epidemic of infectious diarrhea among 13 cities in Jiangsu, which reminds us that in cold seasons, more preventive strategies and measures should be done to prevent infectious diarrhea.

**Electronic supplementary material:**

The online version of this article (10.1007/s11596-021-2338-x) contains supplementary material, which is available to authorized users.

Infectious diarrhea is one of the major public health problems worldwidely, especially in developing countries. It usually shows a symptom of gastrointestinal infection, which could be caused by bacteria, viruses, or parasites. Infectious diarrhea has been classified as one of the Class C legal infectious diseases in China. Though the morbidity and mortality of infectious diarrhea in children declined over the past two decades, it remains a major contribution to the burden of children's diseases[1]. Studies have shown that 72% of diarrheal deaths occurred in children under two years old globally[1]. In Africa, children under five years old experience an average of at least three diarrheal episodes every year[2]. A meta-analysis of nine studies showed a probability of growth retardation attributed to diarrhea was 1.13 (95% CI, 1.07–1.19) for children under two years of age[1, 3]. Etiologically, virus infections, especially norovirus or rotavirus infections in cold seasons account for about 99.7% cases of infectious diarrhea among children[4]. Since its high prevalence incidence, infectious diarrhea poses a deleterious effect on children's health, and unfortunately there is no specific vaccine available to prevent it yet.

Many studies have shown that meteorological factors are closely related to infectious diseases.

Nan-nan HUANG, E-mail: 220193573@seu.edu.cn; Hao ZHENG, E-mail: zhenghao@jscd.cn
[†]Both authors contributed equally to this study.
[#]Corresponding author, E-mail: 101011116@seu.edu.cn
**Electronic supplementary material** The online version of this article (https://doi.org/10.1007/s11596-021-2338-x) contains supplementary material, which is available to authorized users.

Alterations in maximum and minimum temperatures, drought or rainfall, air and water quality could remarkably increase the morbidity and mortality of infectious diseases[5–7]. Specifically, the influence of temperature on diarrhea-related morbidity mainly occurs in childhood (0–14 years old) and the elderly (40–64 years old) instead of adolescents and adults (15–39 years old)[8]. An investigation conducted in Netherland showed a high proportion of susceptible people and low temperature were associated with the accelerated spread of infectious diarrhea in 2014[9]. Zhou *et al* identified maximum temperature and rainfall was strongly linked to diarrhea-associated morbidity in Taiwan[10]. Greer *et al* reported that for each degree centigrade rising in temperature, norovirus infection risk was increased by 8% in Canada[11]. Reena *et al* suggested that global climate change enhanced the incidence of infectious diarrhea in the Pacific Islands[12]. Researches targeting on the relationship between infectious diarrhea and climatic factors are required.

In the present study, we aimed to quantitatively examine the effects of meteorological factors on the prevalence of infectious diarrhea by using the related data of 13 cities in Jiangsu, China from 2015 to 2019. The effects of meteorological factors on diarrhea might be nonlinear and delayed[13–15]. Therefore, quasi-Poisson regressions with distributed-lag nonlinear model (DLNM), which can estimate the lag-effect of meteorological factors on infectious disease, were required to explore the association between meteorological factors and infectious diarrhea.

## 1 MATERIALS AND METHODS

### 1.1 Research Area

This study included infectious diarrhea cases that occurred in 13 cities of Jiangsu Province from January 1, 2015 to December 31, 2019. The temperature in Jiangsu Province decreases from southern to northern areas. For example, the average daily temperatures for each day of the year in Jiangsu Province ranged from 13°C to16°C in the South of the Yangtze River, 14°C to 15°C in the Yangtze River basin, and 13°C to 14°C in Huaibei and coastal areas, rising gradually from northeast to southwest. The lowest temperature occurs in January, ranging from –1.0 to –3.3°C, and in July, the hottest month, the average temperature changes between 26°C and 28.8°C.

### 1.2 Data Collection

All the infectious diarrhea cases were defined based on the standard clinical diagnosis. Personal data of diarrhea including gender, date of birth, address, and date of onset from January 1, 2015 to December 31, 2019 were collected by Jiangsu Center for Disease Control and Prevention (Jiangsu CDC). Daily numbers of diarrheal cases were calculated and further used in this study. Annual demographic data of each city were obtained from the Statistical Yearbook of Jiangsu Province (http://tj.jiangsu.gov.cn/col/col76362/index.html). Daily meteorological data were acquired from the China's National Meteorological Information Center (https://data.cma.cn).

The meteorological variables included daily maximum temperature (Tmax), daily minimum temperature (Tmin), daily average temperature difference (MTD), daily average air pressure (HPA), daily average relative humidity (RH), and daily precipitation (MP). We calculated the average level of each meteorological variable in each city using monitoring data from more than one meteorological station.

### 1.3 Statistical Analysis

**1.3.1 Descriptive Analysis** Major demographic characteristics, the time series distribution of meteorological factors and infectious diarrhea among children aged 0–5 years old (including 5 years old) in Jiangsu Province from January 1, 2015 to December 31, 2019, were described in this study. In addition, the correlations between meteorological factors (HPA, Tmax, Tmin, RH, MP, and cases of infectious diarrhea each day were explored by using the Spearman correlation analysis.

**1.3.2 The Nonlinear and Lagged Relationship between Ambient Temperature and Infectious Diarrhea** We assumed that the daily counts of infectious diarrhea followed a quasi-Poisson distribution since it is a small probability event and may have the over-dispersion problem. In addition, in order to deal with the potential incubation period of infectious diseases, a conditional Poisson regression in combination with the DLNM[16] (http://www.ag-myresearch.com/package-dlnm) was used in this study. The DLNM provides a powerful method to assess nonlinear and lagged effects simultaneously, and it is quite suitable to explore the nonlinear relationship between meteorological factors and health. The formula we used in this study is as follows:

$$\text{Log}\,[E(Yt)] = \alpha + cb(\text{Tmax, lag}) + ns\,(\text{RH}) + ns\,(\text{MP}) + ns\,(\text{time}) + \text{DOW}$$

where Log [E (Yt)] is interpreted as the expected numbers of infectious diarrhea on day t, α indicates intercepts, cb (Tmax, lag) explains the cross-basis function for maximum temperature (Tmax), which generates the basic matrices for exposure-response as well as lag-response relationships in the two dimensions, ns (RH, lag) denotes the natural spline function for confounding factor daily average relative humidity (RH), ns (MP) presents the natural spline function for confounding factor daily precipitation (MP), ns (time) denotes the natural spline function to control for long-term trends and seasonality (seven df per year), and DOW refers to the day of the week effect. Data management, data analyses and graphics were performed in R version 4.0.2 using stats" (https://github.com/StatsWithR/statsr), "tsModel" (https://CRAN.R-project.org/package=tsModel), "MASS" (http://www.stats.ox.ac.uk/pub/MASS4/) and "DLNM" packages.

A "J", "U", or "V" shape exposure response relationship between ambient temperature and health outcomes[17–19] has been found in many studies on ambient temperature and health, which means an "optimum temperature threshold or interval" might occur. In this study, we set the reference value of "optimum temperature threshold" for infectious diarrhea as 16.7°C according to previous studies[20] and calculated the relative risks (RRs) for lagged day from 0 to 21 in each city.

## 2 RESULTS

### 2.1 Descriptive Analysis of Infectious Diarrhea and Meteorological Factors in Jiangsu Province from January 1, 2015 to December 31, 2019

Daily incidence of infectious diarrhea during the study period was 0.443 cases per million people in Jiangsu Province, and it ranged from 0.048 to 0.813 per day among 13 cities with the highest incidence in Suzhou (0.8133/million) and the lowest incidence in Suqian (0.0483/million) (table 1). As shown in fig. 1, the incidence of infectious diarrhea in children under 5 years of age was seasonal and periodic, and the most daily cases of infectious diarrhea occurred in winter, from November to February each year. The daily number of diarrheal cases in winter of 2018 and 2019 was higher than that in the previous three years, with the highest number in 2018 (13219 cases). A temporal reverse trend was found between the daily incidence of infectious diarrhea and the daily Tmax (fig. 1). However, similar trends were not found between the daily incidence of infectious diarrhea and other meteorological factors (fig. S1).

**Table 1 The number and daily incidence of infectious diarrhea among 13 cities in Jiangsu Province**

| City | The number of infectious diarrhea | Daily incidence of infectious diarrhea (per million) |
|---|---|---|
| Nanjing | 6747 | 0.445846 |
| Wuxi | 6853 | 0.573564 |
| Xuzhou | 8555 | 0.53828 |
| Changzhou | 1853 | 0.21485 |
| Suzhou | 15830 | 0.813331 |
| Nantong | 2755 | 0.206575 |
| Lianyungang | 2034 | 0.248508 |
| Huaian | 1332 | 0.149402 |
| Yancheng | 9814 | 0.743338 |
| Yangzhou | 699 | 0.085108 |
| Zhengjiang | 4042 | 0.695257 |
| Taizhou | 3910 | 0.460914 |
| Suqian | 431 | 0.048302 |

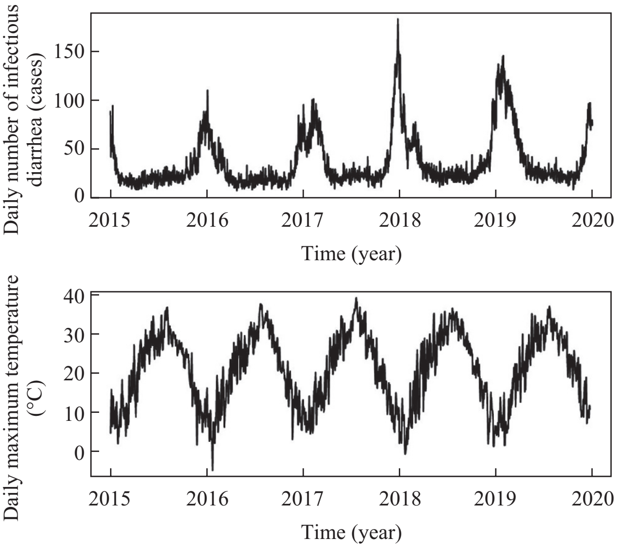

**Fig. 1** Seasonal and monthly distribution of daily cases of infectious diarrhea among children under five years old and daily maximum temperature (Tmax) in Jiangsu Province, China (2015–2019).

A total of 62 729 cases of infectious diarrhea were reported in children under 5 years old (including 5 years old), and the ratio of male to female was 1.54:1 from January 1, 2015 to December 31, 2019. As shown in fig. 2, the subjects were divided into groups "≤ 1", "1 < age ≤ 2", "2 < age ≤ 3", "3 < age ≤ 4", and "4 < age ≤ 5" according to age. Male cases were consistently more than females among five age groups, especially in the 0–2 year age group. Results showed that age group "≤ 1" had the highest number of infectious diarrhea, accounting for 82% of the total cases. Group "1 < age ≤ 2" accounted for 13% of the total number of infectious diarrhea. The numbers of infectious diarrhea cases decreased with age increasing.

The spatial distribution of reported infectious diarrhea cases is shown in fig. 3. Suzhou has the highest number of reported cases from 2015 to 2019 (15 803 cases), accounting for 24% of the total reported cases, followed by Yancheng, Xuzhou, Nanjing, and Wuxi (15%, 13%, 11% and 10% of the total reported cases,

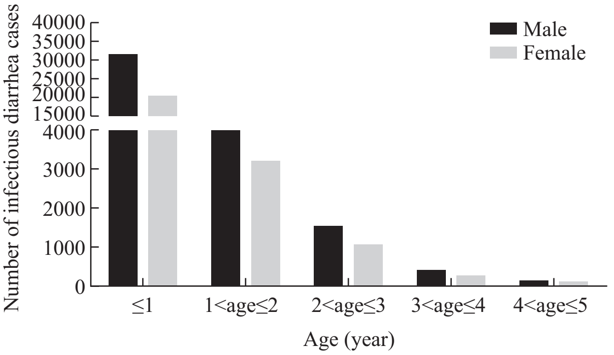

**Fig. 2** Age and sex distribution of number of infectious diarrhea cases in Jiangsu Province, China (2015–2019).

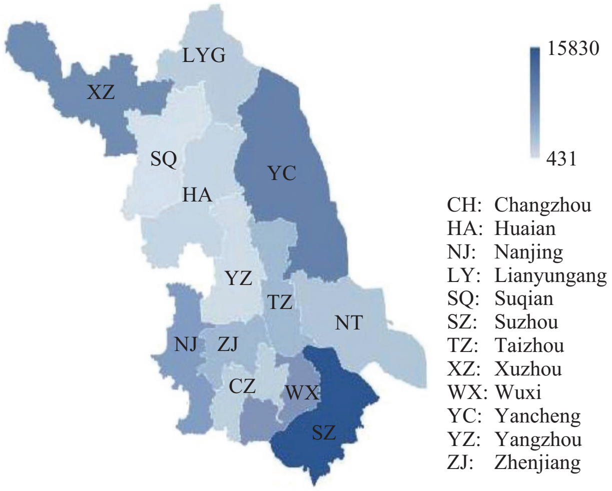

**Fig. 3** Regional distribution of daily cases of infectious diarrhea in Jiangsu Province, China (2015–2019).

respectively). Yangzhou and Suqian had a relatively low proportion (1.0% and 0.7%, respectively) of total cases.

### 2.2 Correlations between Infectious Diarrhea and Meteorological Factors

Spearman correlation analysis showed a statistically significant correlation (*P*<0.0001) between the daily number of infectious diarrhea (N) and HPA, Tmax, Tmin, RH, or MP except MTD (fig. 4). The coefficients of HPA with Tmax, Tmin were –0.87, –0.89 (*P*<0.05), respectively. In addition, according to the collinearity analysis in table S1 and fig. 4 and the strong association of RH with temperature effects, only Tmax, MP and RH were included into Poisson's regression model combined with DLNM. When the reference value was set at 16.7°C, the relative risks (RRs) for lagged day from 0 to 21 in each city are shown in fig. 5. Effects of Tmax on infectious diarrhea varied from 13 cities and the significant lag-effect was observed in 8 cities (RR>1 and *P*<0.05) including Nanjing, Wuxi, Changzhou, Lianyungang, Suzhou, Yangzhou, Huaian, and Suqian. The highest RR value [1.17 (95% CI: 1.07, 1.23)] of Tmax appeared on day of lag-5 in Huaian. As a city with the highest number of cases of infectious diarrhea, the maximum RR of Suzhou was 1.04 (95%, CI: 1.03, 1.05) (table 2). We divided the 21 lag days into three time periods, namely week 1 (0–7), week 2 (8–14), and week 3 (15–21), and found that the lag-effects among 8 cities varied during 21 lagged days. For example, the lag-effect in Changzhou mainly occurred in week 1, while the lag-effects maintained from week 2 to week 3 in Nanjing, Lianyungang, and Suzhou. Generally, the lag-effects of increased risk of infectious diarrhea were found for Tmax. Table 2 and fig. 6 show the associations with Tmax differ between different age groups ("age ≤ 2" *vs.* "2 < age ≤ 5") in Jiangsu. The Maximum RR of Tmax on infectious diarrhea cases in age group "age ≤ 2" was 1.04 (95% CI: 1.03–1.05), while in age group "2 < age ≤ 5" was 1.02 (95% CI: 1.00–10.6). The largest cumulative RR of Tmax on infectious diarrhea cases in age group "2 <age ≤ 5" occurred on lag day 6, while in age group "age ≤ 2" that occurred on lag day 11.

## 3 DISCUSSION

In the present study, we found that the incidence of

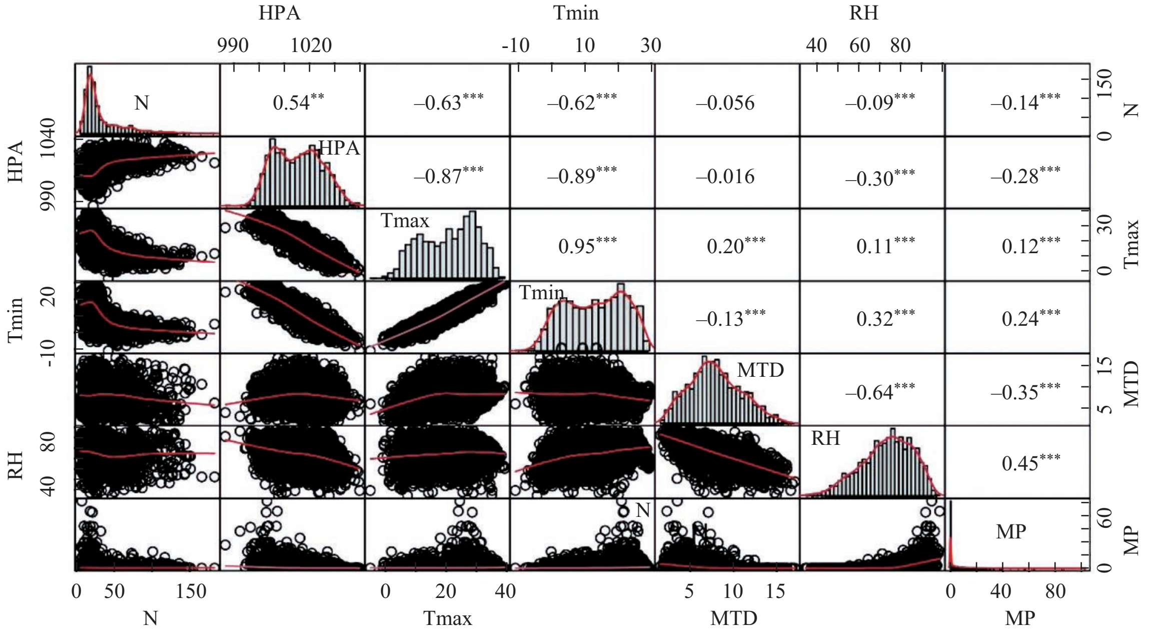

**Fig. 4** Spearman correlation coefficient between meteorological factors and daily cases of infectious diarrhea among children under five years (Tmax: daily maximum temperature, Tmin: daily minimum temperature, MTD: daily average temperature difference, HPA: daily average air pressure, RH: daily average relative humidity, MP: daily precipitation). **P*<0.05, ***P*<0.001, ****P*<0.0001

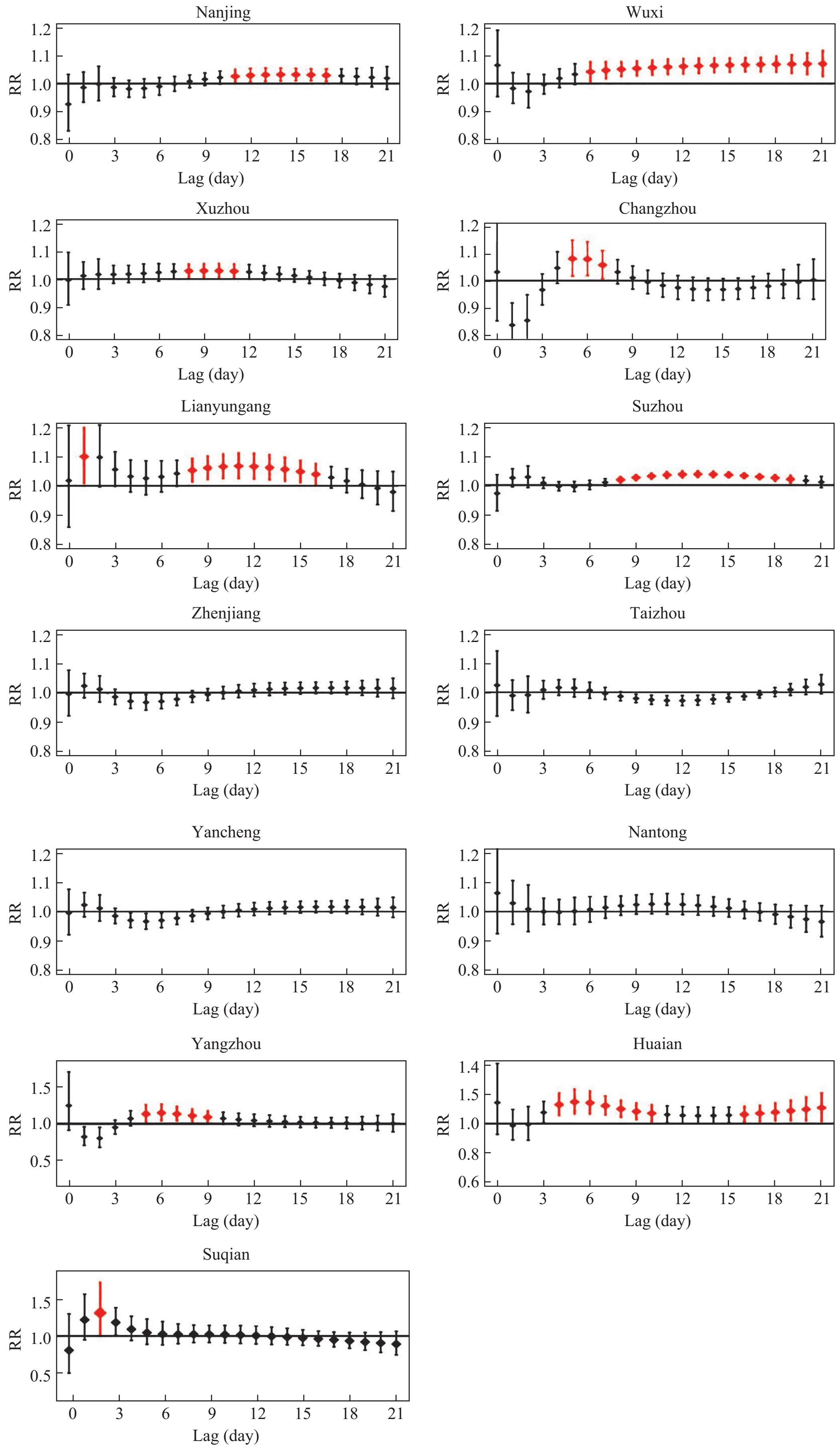

**Fig. 5** RR estimates of the Tmax on the risk of daily cases of infectious diarrhea among children under five years in different lag days among 13 cities in Jiangsu Province, China (lag0–21)

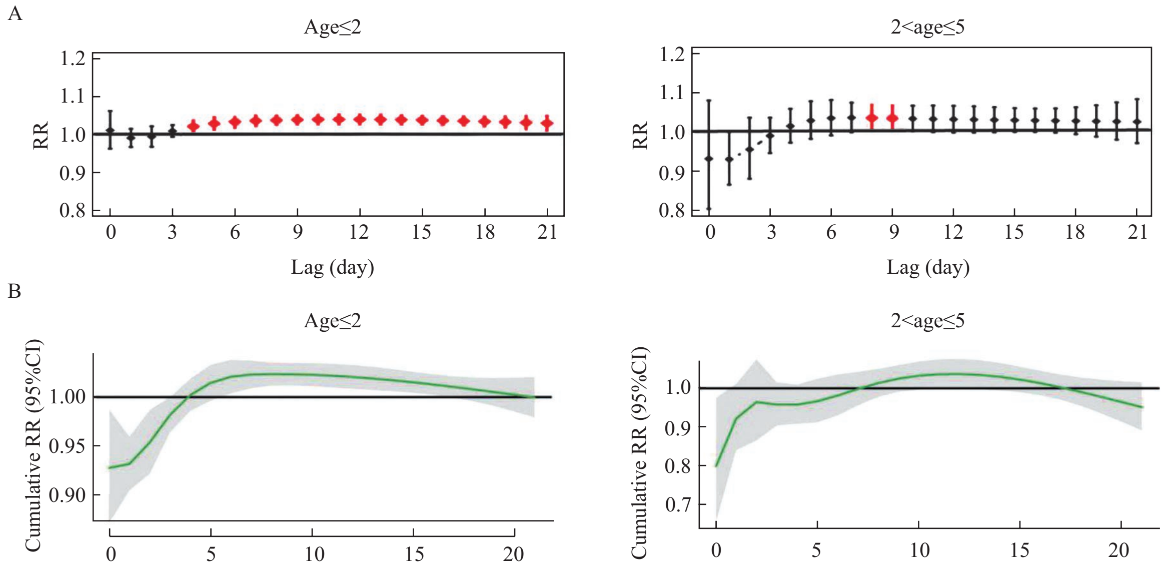

**Fig. 6** Estimation of the Tmax on the risk of daily cases of infectious diarrhea among children in age groups (“age≤2” *vs.* “2<age≤5”) in different lag days in Jiangsu Province, China (lag0–21). A: RR; B: cumulative RR

**Table 2 The largest separate effects of mean daily maximum temperature on infectious diarrhea cases in age groups (“age≤2” *vs.* “2<age≤5”) and city groups in Jiangsu Province**

| | Maximum RR (95% CI) | Temperature (℃) | Lag (day) |
|---|---|---|---|
| Age group | | | |
| age≤2 | 1.04 (1.03–1.05) | 8 | 11 |
| 2<age≤5 | 1.02 (1.00–10.6) | 8 | 9 |
| City group | | | |
| Nanjing | 1.10 (1.03–1.17) | 8 | 6 |
| Wuxi | 1.07 (1.04–1.09) | 8 | 6 |
| Xuzhou | 1.03 (1.00–1.06) | 8 | 9 |
| Changzhou | 1.10 (1.03–1.18) | 8 | 5 |
| Lianyungang | 1.08 (1.03–1.14) | 8 | 10 |
| Suzhou | 1.04 (1.03–1.05) | 8 | 15 |
| Yangzhou | 1.17 (1.06–1.28) | 8 | 7 |
| Huaian | 1.18 (1.09–1.29) | 8 | 5 |
| Suqian | 1.09 (1.04–1.29) | 8 | 9 |
| Yancheng | 1.02 (0.98–1.07) | 8 | 1 |
| Zhenjiang | 1.02 (0.98–1.07) | 8 | 1 |
| Yangzhou | 1.26 (0.92–1.72) | 8 | 0 |
| Taizhou | 1.03 (0.99–1.06) | 8 | 21 |

infectious diarrhea in Jiangsu Province has a peak period in autumn and winter every year. Studies have shown that the etiology of the infectious diarrhea in autumn and winter was mainly rotavirus and norovirus[21–23] and the virus detection rates were 15% and 12% in China, respectively. Rotavirus is characterized by seasonal infection, and its high incidence in autumn and winter is the most frequent cause of fatal diarrhea of children under five years old worldwide[8, 9].

We found that the incidences of infectious diarrhea were higher in younger age groups, especially in those under 1 year of age, which reminds us babies under 1 year old were more vulnerable to infectious diarrhea, which could bring more detrimental impacts on children’s growth and development. It has been reported that the incidence of diarrhea episodes increased the relative risk (RR) of growth retardation by 1.13 (95% CI 1.07–1.19) in infants and young children[3]. In addition, children in age group “age≤2” was more vulnerable to Tmax than those in age group “2<age≤5”. Zhou *et al* have shown that higher temperatures were associated with the epidemic of diarrhea disease by viral exposure and transmission for diarrhea in younger group. This may be due to the imperfect development of the immune system, poor gastrointestinal function, and immune deficits relative to intestinal pathogens in infants. Moreover, the age and the education of the mother, the type of toilet facilities, non-exclusive breastfeeding and bottle feeding were also risk factors for diarrhea in children[24]. A clinical trial showed a significant reduction in parasite detection rates following water, sanitation, and hygiene interventions[25, 26]. Fang *et al* had reported three high endemic regions of infectious diarrhea in Jiangsu Province from 2013 to 2017, which were Xuzhou, Yancheng and the border area between Wuxi and Suzhou[27]. In our study, we found that the incidences of infectious diarrhea in Xuzhou and Yancheng were decreased during 2015 to 2019; however, the highest incidence was still identified in Suzhou from 2015 to 2019. The high population density and the large number of non-resident population in Suzhou contributed to the disease transmission, especially through the respiratory and fecal-oral routes[28, 29]. On the other hand, infectious diarrhea are self-limited diseases, and many people do not go to hospitals even they have symptoms of diarrhea. Suzhou is one of the high-income areas in Jiangsu, people in Suzhou have better access to medical facilities due to the improved development of primary health care. Therefore, the diagnosis rate of infectious disease there can be higher than that in other areas.

Meteorological factors could profoundly affect infectious diarrheas. In this study, the association between meteorological factors and the number of infectious diarrhea was evaluated. Wang *et al* reported that RH and MTD were strongly associated with the incidence of infectious diarrhea in Guangzhou, China from 2006 to 2017[30]; however, our results found no statistically significant correlation of RH ($r$=–0.09) and MTD ($r$=–0.056) with the number of infectious diarrhea cases. The contrary conclusions can be attributed to the difference in meteorological characteristics between Guangzhou (tropical area) and Jiangsu Province (temperate to subtropical area). Even in Jiangsu Province, the effects of meteorological indicators on the case number of infectious diarrhea varied, depending on the climate characteristics of each city. Compared with average temperature, maximum temperature has been increasingly used by epidemiologists in recent years as a suitable index of temperature[14, 31]. Therefore, we only explored the effect of Tmax, without Tmin, on infectious diarrhea in our DLNM analysis, because the colinearity between Tmax and Tmin. The Tmax ($r$=–0.63, $P$<0.05) for the highest cumulative RR of infectious diarrhea was at 8°C.

Infectious diarrhea is caused by a variety of pathogens, and the incubation period varies based upon pathogens. For example, Lee *et al* reported that incubation periods of astrovirus, norovirus and rotavirus were 4.5 days (95% CI 3.9–5.2 days), 1.2 days (95% CI 1.1–1.2 days), 1.7 days (95% CI 1.5–1.8 days), and 2.0 days (95% CI 1.4–2.4 days), respectively[32]. Meanwhile, compared with adults, the incubation period could be longer for susceptible population such as children[33]. Consistent with the previous studies, our results suggested that an incubation period of infectious diarrhea in children under five years old could be as long as up to 3 weeks. Previous studies have suggested that the lag-effects of Tmax occurring in the first week could be a result of immediate exposure to meteorological factors (such as flood water and temperature), and/or short incubation periods for the infectious diarrhea; further, the lag-effects occurred in the second and/or third week were associated with the indirect exposure on population displacement, drinking water, contaminated food or infections with long incubation periods[34, 35]. An *in vitro* study reported that murine norovirus and group A bovine rotavirus existing in human urine still showed infectious capacity at 15°C and 25°C over an incubation period of 42 days and 49 days, respectively[36]. Therefore, improvement of hygiene in the susceptible population in cold days plays a key role in preventing infectious diarrhea.

A limitation should be mentioned that the pathogens causing the infectious diarrhea were not differentiated since the data on the pathogens of infectious diarrhea were not obtained in this study; therefore, the effects of meteorological factors on specific pathogens remain still unclear.

In a conclusion, our results identified that the prevalence of infectious diarrhea in children under five years old in Jiangsu, China was associated with meteorological factors. Moreover, the lag-effects of Tmax were significantly negatively associated with the occurrence of infectious diarrhea, implicating the shift of preventive intervention beginning from winter to the end of autumn season.

**Open Access**

This article is licensed under a Creative Commons Attribution 4.0 International License https://creativecommons.org/licenses/by/4.0/), which permits use, sharing, adaptation, distribution and reproduction in any medium or format, as long as you give appropriate credit to the original author(s) and the source, provide a link to the Creative Commons licence, and indicate if changes were made. The images or other third party material in this article are included in the article's Creative Commons licence, unless indicated otherwise in a credit line to the material. If material is not included in the article's Creative Commons licence and your intended use is not permitted by statutory regulation or exceeds the permitted use, you will need to obtain permission directly from the copyright holder. To view a copy of this licence, visit http://creativecommons.org/licenses/by/4.0/.

**Conflict of Interest Statement**

The authors declare that there is no conflict of interest with any financial organization or corporation or individual that can inappropriately influence this work.

## Electronic supplementary material

Supplementary material, approximately 25.7 MB.
