# Supplementary material for: The Short-term Effects of Temperature on Infectious Diarrhea among Children under 5 Years Old in Jiangsu, China: A Time-series Study (2015–2019)
Source: Curr Med Sci. 2021 Apr 20;41(2):211–8. doi: 10.1007/s11596-021-2338-x (PMC8056199; doi:10.1007/s11596-021-2338-x)
Supplement: Supplementary file 1 — Supplementary material, approximately 25.7 MB. [file 11596_2021_2338_MOESM1_ESM.pdf]

Table S1 The collinearity analysis between meteorological factors in Jiangsu Province, China (2015–2019)

City	HPA[a]	Tmax[a]	Tmin[a]	MP[a]
VIF[b]	5.59289	18.2135	18.3326	1.34408

HPA: daily average air press; Tmax: daily maximum temperature; Tmin: daily minimum temperature; MP: daily precipitation; VIF: variance inflation factor

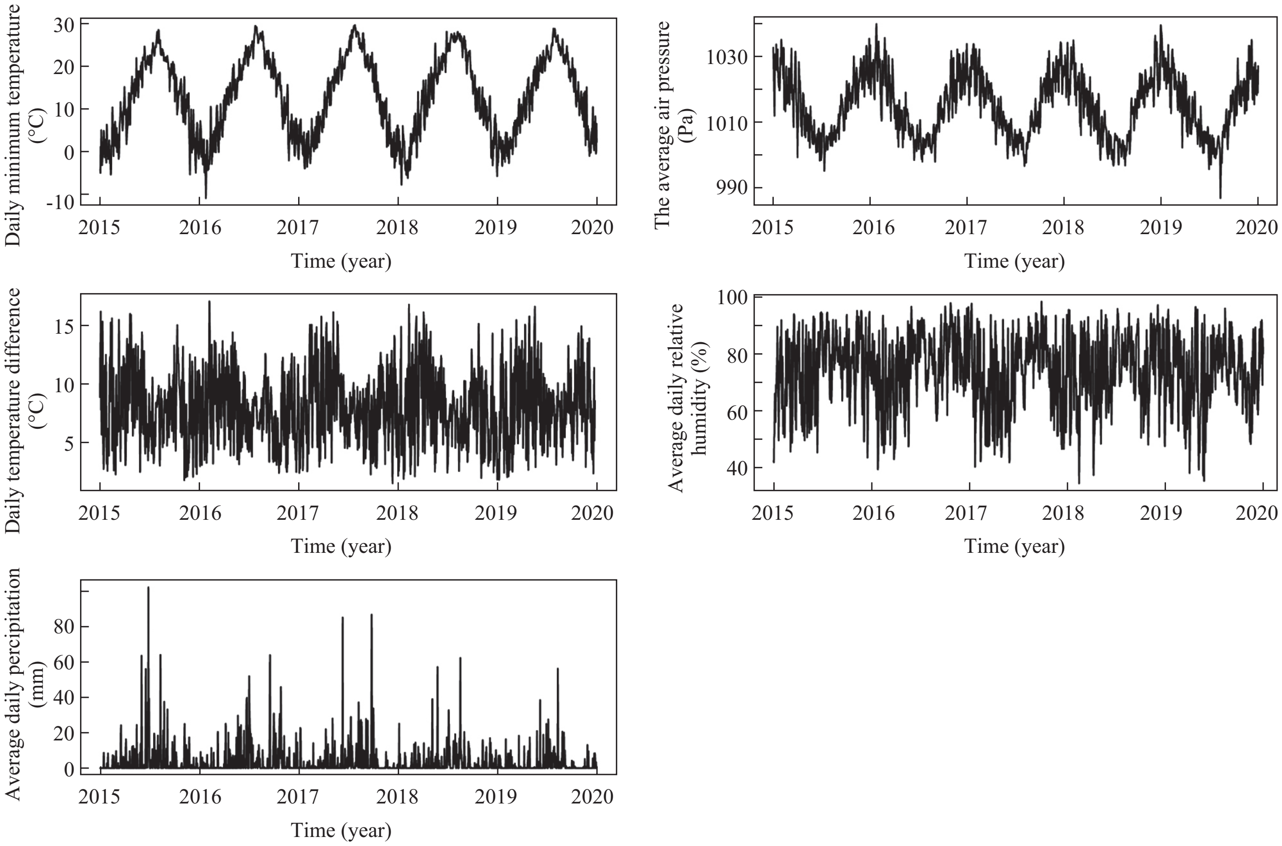

Supplemental Fig. 1 Seasonal and monthly distribution of meteorological factors in Jiangsu Province, China (2015–2019)
Tmin: daily minimum temperature; MTD: daily average temperature difference; HPA: daily average air press; RH: daily average relative humidity; MP: daily precipitation

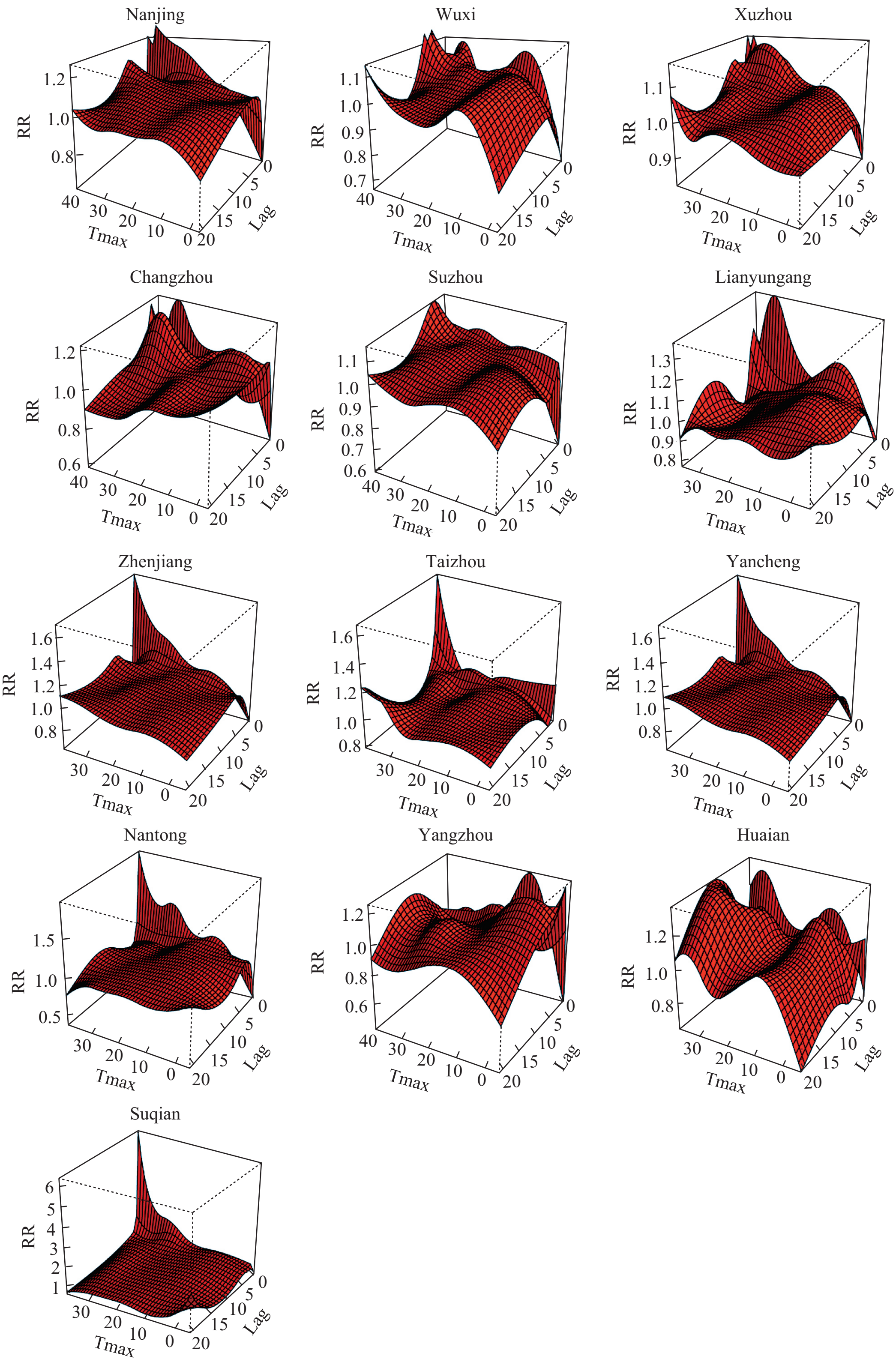

Supplemental Fig. 2 3D plots of relative risks of Tmax on infectious diarrhea within 21 lag day
